# A New Dawn for Brazilian Pediatric Cardiac Surgery Is on the Way - Issues Around and Outside the Operating Room

**DOI:** 10.21470/1678-9741-2022-0141

**Published:** 2022

**Authors:** Luiz Fernando Caneo, Leonardo Augusto Miana, Daniel Garros, Rodolfo Neirotti

**Affiliations:** 1 Pediatric Cardiac Surgery Unit, Cardiovascular Division, Instituto do Coração, Faculdade de Medicina da Universidade de São Paulo, São Paulo, São Paulo, Brazil; 2 Department of Pediatrics, Division of Critical Care, Faculty of Medicine, University of Alberta, Stollery Children’s Hospital, Edmonton, Alberta, Canada; 3 Pediatric Cardiovascular Surgery Department, Sociedade Brasileira de Cirurgia Cardiovascular (SBCCV), São Paulo, São Paulo, Brazil; 4 Clinical Professor of Surgery and Pediatrics, Emeritus Michigan State University, Michigan, United States of America

**Keywords:** Cardiac Surgery, Congenital Heart Defects, Developing Countries, Children, Public Health Administration, Cause of Death.

## Abstract

In some developing countries, congenital heart disease still stands out among the leading causes of death in the first year of life. Therefore, there is a great need to develop programs designed to improve outcomes in the diagnosis and surgical treatment of congenital heart disease in these nations, where children have always been and still are severely underserved.

The Brazilian Public Health Care System demands universal access to treatment as a constitutional right. Therefore, an underfunded Pediatric Cardiac Surgery program is unacceptable since it will cost lives and increase the infant mortality rate. Additionally, poor funding decreases providers’ interest, impedes technological advances and multidisciplinary engagement, and reduces access to comprehensive care.

Unfortunately, in most developing countries, Pediatric Cardiac Surgery progress is still the result of isolated personal efforts, dedication, and individual resilience. This article aims to present the current state of Brazilian pediatric cardiac surgery and discuss the structural and human limitations in developing a quality care system for children with congenital heart disease. Considering such constraints, quality improvement programs via International collaboration with centers of excellence, based on proper data collection and outcomes analysis, have been introduced in the country. Such initiatives should bring a new dawn to Brazilian Pediatric Cardiac Surgery.

**Table:** 

Abbreviations, Acronyms & Symbols			
CHD	= Congenital heart disease	LOS	= Length of stay
CHL	= Children’s HeartLink	MG	= Minas Gerais
CPB	= Cardiopulmonary bypass	NGOs	= Non-governmental organizations
ECMO	= Extracorporeal membrane oxygenation	PCICU	= Pediatric cardiac intensive care unit
GDP	= Gross domestic product	PCS	= Pediatric Cardiac Surgery
InCor-HC-FMUSP	= Instituto do Coração do Hospital das Clínicas da Faculdade de Medicina da Universidade de São Paulo	PR R$ RN	= Paraná = Brazilian Real = Rio Grande do Norte
HLMs	= Heart-lung machines	RS	= Rio Grande do Sul
ICU	= Intensive care unit	SBCCV	= Sociedade Brasileira de Cirurgia Cardiovascular
IMR	= Infant Mortality Rate	SJRP	= São José do Rio Preto
InCor	= Instituto do Coração	SP	= São Paulo
IQIC	= The International Quality Improvement Collaborative	SUS TPS	= Sistema Único de Saúde = Technical Performance Score
LMICs	= Low-income and middle-income countries		

## INTRODUCTION

Health care systems are multidimensional and are significantly affected by socioeconomic, cultural, and political factors, particularly in the developing world. This reality is very palpable in Brazilian Pediatric Cardiac Surgery (PCS). Political and economic crises are certainly an impediment to adapting to new technologies and new ways of thinking. In Latin America, with rare exceptions, most programs that offer heart surgery for children suffer from stagnation and continue with suboptimal outcomes^[1^-^3]^.

Conversely, developed countries differentiate themselves based not only on more financial resources but also on dealing with the unforeseen problems of complex systems. It has been said that creativity allied with technology can eventually help Latin America to progress where disparities and unfairness are undeniable^[4]^.

Altogether, we face a situation that justifies further efforts to improve outcomes if we follow the message of Machado’s manuscript that states: “As administrators, we need to consider the best way to protect as many lives as we can”^[5]^.

With a critical view of the current reality, the purpose of this article is to show that we are pursuing a new tomorrow for PCS in Brazil, especially after understanding our strengths, weaknesses, opportunities, and threats in caring for children with congenital heart disease (CHD) in our country.

## OVERVIEW

When discussing PCS, we must remember that, as a large country, Brazil has a heterogeneous scenario regarding the number of surgeries performed, centers’ distribution, and hospital resources. Brazil is working to improve organizational, human, and financial resources, and as an “emerging country”, it has the opportunity and openness for international cooperation. Brazilian pediatric cardiac care is gradually moving from a “personality-centered” to an “institutional-centered” outcomes-focused approach, investing in better teamwork, international collaboration, and better data mining to achieve better results.However, the challenges are enormous to acquire the infrastructure needed to reach higher performance.

The strength of this manuscript is the authors’ empiric knowledge of the reality of PCS programs in Brazil due to their multiple levels of involvement in research and active participation in the Brazilian cardiac surgery societies over many years. It is not meant to be a comprehensive report about the status quo of cardiac surgery programs in the country.

## PEDIATRIC CARDIAC SURGERY IN THE BRAZILIAN PUBLIC HEALTH CARE SYSTEM

### A. The Status Quo

Brazil is the largest country in South America, with around 220 million inhabitants. It is estimated that the prevalence of CHD is between 7-10 per 1000 births^[6]^. Considering that the country’s live births were 2,989,981 per year in the last decade, we can infer that Brazil has an influx of 28,846 children with CHD per year^[7]^. Furthermore, in approximately 20% of these cases, the defects resolve spontaneously, or the malformation is less complex and carries discrete hemodynamic repercussions^[2]^. Based on these parameters, at least 24,000 procedures/year would be necessary for Brazil to meet the CHD children’s needs, without mentioning reoperations for complications^[2^,^6^,^8^-^9]^.

The reality is that a median of 9,000 surgeries/year was registered in the Brazilian Sistema Único de Saúde (SUS) (our Unified Health System) database (or DATASUS) from 2010 to 2020. In 2017, a new governmental program was launched to stimulate a surge in the number of surgeries performed annually, the “Plano Nacional de Assistência à Criança com Cardiopatia Congênita” (or National Assistance Plan for Children with CHD). In addition, the SUS remuneration table for PCS was modified to increase the number of recognizable procedures to 49, according to case complexity. This action represented a growth of 75.2% of the original budget dedicated to the specialty in the public system. This initiative, coordinated by the Fundo de Ações Estratégicas e Compensação (or FAEC, Fund for Strategic Actions and Compensation), has granted sufficient funds to support at least an increase of 30% in the number of procedures. That would represent more than 3,400 hospital procedures per year.

**Fig. 1:**
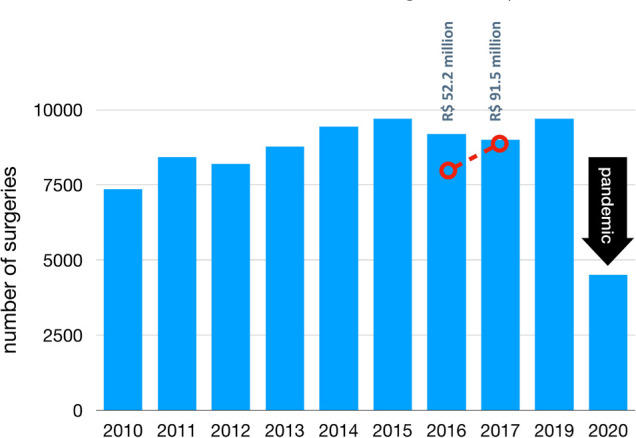
Incremental number of pediatric cardiac surgeries over one year by government funding enhancement, with the 2020 reduction due to the pandemic. Blue bars represent the number of procedures each year and the red dotted line represents the government budget. R$=Brazilian Real.

Unfortunately, in the following two years, the surgeries performed were only 59% of the Ministry of Health’s expectations due to several constraints (Figure 1). Furthermore, in 2020, the coronavirus disease 2019 (or COVID-19) pandemic severely affected all Brazilian CHD Surgery programs by reducing surgical volume, unbalancing case-mix towards more complex cases, and consequently increasing mortality rates^[10]^.

Although it should be a constitutional right, universal and comprehensive care is not a reality in Brazil due to its continental dimensions, large and unevenly distributed population, and the patchy geographical distribution of pediatric cardiology and adult cardiology surgery centers.

Despite the government, medical societies, and surgeons’ efforts, there is no consensus on solving several problems that limit the development of a solid and inclusive PCS system in the country. One fundamental aspect is the lack of reliable data, making it challenging to understand the exact scenario of caring for children with CHD.

In 2019, a government-funded nationwide study was performed with *in situ* visits carried out by the Brazilian Society of Cardiovascular Surgery surgeons. The project collected information from 54 out of 67 hospitals registered as pediatric cardiac centers, and 12 out of the 13 centers that refused the visit are not currently performing PCS procedures. Although we lack information from insurance-covered surgeries in totally private facilities that correspond to around 10-15% of the total number of procedures, we could closely estimate the number of procedures performed before 2019. Altogether, the information obtained by this surveillance has helped us to substantiate the arguments presented here regarding the status of CHD surgery in Brazil, focusing on its strengths, weakness, opportunities, and threats.

### B. Historical Context

PCS has been offered for many years in Brazil, thanks to pioneers’ creativity and hard work, *i.e.*, some individuals who were able to produce good work despite limited resources. Leadership, patience, perseverance, dedication, and the capacity to adapt to adversity have been the keys to success during the earlier days. The innovators had the opportunity to train abroad with world-renowned pioneers in the field. After returning to Brazil, they built a “surgical school” in the Southeast region that eventually placed cardiovascular surgery ahead of its time. The scientific contributions and developments in techniques in pediatric surgery that have appeared in the world literature over the years are good examples of the creativity, expertise, and innovation of the Brazilian pioneer surgeons attracting local and international attention^[11]^.

Their resilient history of local innovation in the earliest days of general cardiac surgery encompasses advances in heart-lung machines (HLMs), cardiac valves, conduit implants, and new surgical techniques. For example, in 1959, Brazil started to produce its own HLMs and used one of them to perform the first adult heart transplantation in South America^[12]^. These advances highlighted the collaboration and teamwork between surgeons and biomedical engineers. Interestingly, surgeons and other physicians were the first perfusionists. Furthermore, perfusion products, including different oxygenators, were developed and manufactured domestically.

It was up to the late 1970s when most of the new developments in PCS worldwide moved from centers doing both adult and pediatric operations to children’s hospitals with exclusive PCS programs. By 1975, 10% of the patient’s procedures were performed within the first six months, and 19% were within the first 12 months of life. Around 60% of the children are operated on in their first year of life, and 30% in their first month. This trend - the movement of surgery toward the very young - has developed because of significant advances in understanding neonate and infant physiology. That approach has numerous biological and socioeconomic benefits. The excellent outcomes obtained, particularly in specialized PCS centers, directly result from an ongoing generation of knowledge and partnership between pediatric cardiologists, cardiovascular surgeons, and intensivists^[13]^.

In the beginning, and still now, cardiac surgeons worked in congenital and acquired cardiovascular diseases in Brazil. Initially, a “heart center model”, where adult cardiac surgery and PCS were performed under one roof, was adopted in most centers in our country. But, regrettably, the distance between the heart centers and designated children’s hospitals compromised not only the development of neonatal cardiac care but also explains the delay of proper implementation of complex therapies like extracorporeal membrane oxygenation (ECMO) - an important tool to manage respiratory distress due to meconium aspiration and post-cardiotomy shock after complex cardiac procedures under bypass.

Our pediatric cardiac centers were finally trained and stimulated to enhance ECMO adoption over the last ten years through partnerships with specialists and institutions from the developed world. The foundation of the Latin America chapter of the Extracorporeal Life Support Organization (or ELSO) in 2012 consolidated such partnerships, establishing locally organized multidisciplinary training programs following international standards. The first ECMO Specialist Training Course^[14]^ was held in Brazil in 2013 at the Instituto do Coração (InCor), Faculdade de Medicina da Universidade de São Paulo, with the collaboration of the ECMO team from the Stollery Children’s Hospital, Edmonton, Canada. The training has wholly changed ECMO history in Brazil by preparing pediatric cardiac centers that subsequently demonstrated better outcomes, as shown by Miana LA et al.^[15]^.

**Fig. 2:**
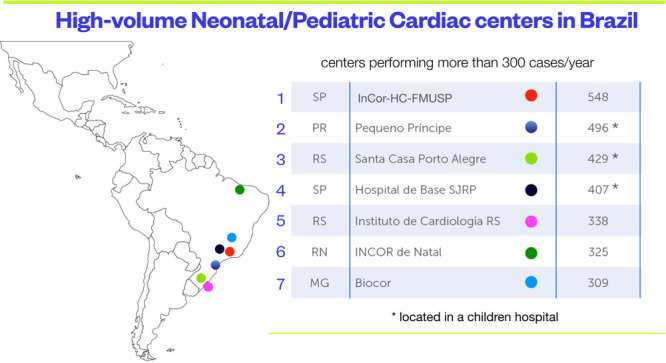
Location and caseload of the largest pediatric cardiac surgery centers in Brazil. InCor-HC-FMUSP=Instituto do Coração do Hospital das Clínicas da Faculdade de Medicina da Universidade de São Paulo; INCOR=Instituto do Coração; MG=Minas Gerais; PR=Paraná; RN=Rio Grande do Norte; RS=Rio Grande do Sul; SJRP=São José do Rio Preto; SP=São Paulo

Another important aspect of this context is that 90% of the Brazilian PCS are still performed in “Heart Centers” or general hospitals rather than in pediatric facilities. The pendulum is maybe swinging back (Figure 2). Recently, the hospital admissions for adults with CHD have increased at a higher proportion than for children, supporting the need to optimize the care for adults. As this drift of increased adult hospitalization is expected to persist in the near future, a new model of cardiac care delivery to CHD patients requires further discussion. Despite being considered by some an outdated model, in our view, the “heart center” model, caring for both adults and children under one roof - together but separated -, seems not to be the main obstacle to develop an adequate pediatric cardiac program in our country.

### C. Training Pediatric Cardiac Surgeons

At the 2005 meeting of the American Board of Thoracic Surgery, a proposal to establish a subspecialty certificate in congenital heart surgery was unanimously approved by the Board of Directors. This proposal was prompted by the recognition that the discipline of congenital heart surgery requires unique skills and education that are not currently provided in a standard cardiothoracic surgery residency. Unfortunately, PCS is still not considered a subspecialty in Brazil. This issue is a critical vacuum that the Sociedade Brasileira de Cirurgia Cardiovascular (SBCCV) should resolve to stimulate a new generation of surgeons to choose this career.

In Brazil, there are very few dedicated PCS training programs, and almost all of them are located in the Southeast region’s high-volume centers.

### D. The Economic Problem: Pediatric Cardiac Surgery with Limited Resources

Limited resources are a constant problem forcing programs to focus on short-term solutions and uncertainty about tomorrow’s needs. A great deal of energy is spent in negotiations to convince people to continue to work hard for low pay, which may partly explain our suboptimal efficiency.

In Brazil, health care is a constitutional right. Inspired by the British National Health Services (or NHS), it represents one of the most comprehensive health care systems in the developing world. The local unified free access system - SUS - is a crucial feature of the 1988 Constitution, drafted at the end of military rule^[16]^.

Nonetheless, the care is provided by both private and government institutions. Primary health care remains the federal government’s responsibility, and individual states oversee some elements, such as hospital care. Brazil is the only country with more than 100 million inhabitants to have a universal health care system, free for all. Without the SUS, 78% of the people who do not have private insurance would be deprived of health services, especially of highly complex procedures, such as PCS.

It gets even worse because cardiac centers are irregularly concentrated in the South and Southeast regions of the country. Of the 67 qualified hospitals performing PCS, 51% are in only four out of 27 Brazilian states. Only six (37.5%) out of 16 North and Northeast states have qualified services for PCS available to their population. Therefore, there is an irregular distribution of procedures, with a higher concentration being proportional to the center’s location and not according to the population’s needs. This inequity is very relevant in such a vast country, considering that traveling long distances with sick children is very expensive and carries a severe risk.

The health system also has a weak structure for regulating hospital beds, making access difficult to the referring centers; this is particularly complicated for those children being followed on an outpatient basis elsewhere. Consequently, only the more severe cases are admitted. Thus, the timing of surgery is tied up to demand, location, and bed availability. This practice selects the more complex cases for admission in the specialized centers occupying the totality of beds available in the system. Due to the case-mix and complexity, most cardiac centers have reduced work capacity because of these complex patients’ longer length of stay (LOS). The government does not financially compensate heart centers treating such complex cases with prolonged intensive care unit (ICU) LOS with any differential payment.

PCS is clearly underfunded, denoting another example of societal lack of priority in using already limited governmental resources. Brazil needs to strengthen its health care system by investing in well-managed and better-distributed institutions that demonstrate efficiency, good management, and evidence-based clinical practices. Moreover, 80% of the government spending goes into social security and payroll, which makes Brazil a total outlier among its peers. Then, it should not come as a surprise that the government investment in health has dropped from a peak of 5.4% of gross domestic product (GDP) 50 years ago to < 1% estimated for this year. Deep reforms are required if the country is willing to mobilize the necessary resources to move away from the longstanding and sad situation of unequal/unsustainable stagnation^[17]^.

Despite all the structural flaws, the SUS has proven to be an affordable provider of widespread health care coverage and a promoter of social equity.

Human resources are affected by the low salaries paid by the medical institutions, which is another factor that may explain the heterogeneous distribution of personnel around the country. Due to the income target model, the government has both places of hyper concentration of professionals coexisting with “medical deserts” in underserved areas.

Doctors with four or more jobs have gone from 24% to 44% in five years. In 2014, 32% said they worked > 60 hours a week; in 2019, this rate reached 46%^[18]^.

**Fig. 3:**
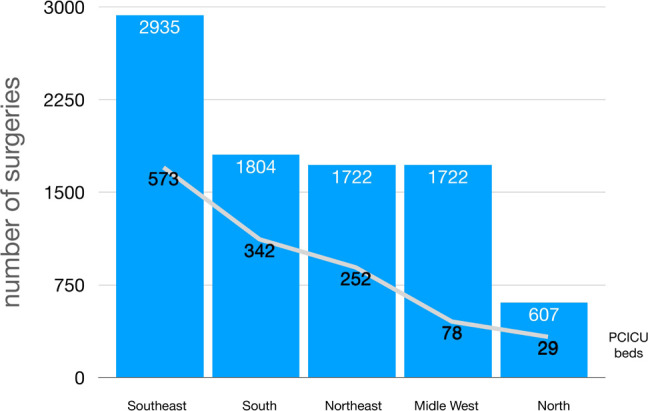
Number of surgeries per region compared to availability of pediatric cardiac intensive care unit (PCICU) beds.

This increased workload can have consequences both for the health of physicians and the quality of service they provide. Most cardiology hospitals lack interest in PCS due to the low pay for the service and the offer of a limited number of beds dedicated to such patients. Our national survey has demonstrated that hospitals with ICU beds dedicated to PCS patients had a more significant number of procedures performed when compared to hospitals without it (Figure 3)^[19]^.

Therefore, the need for dedicated pediatric cardiac ICU beds can be a critical constraint for developing more centers dedicated to CHD patients.

### E. Human Resources: The Multidisciplinary Team

Despite many articles correlating the low number of pediatric surgeons in low-income and middle-income countries (LMICs) to the low number of surgeries performed, this is not a constraint in Brazil^[20]^. According to the SBCCV, Brazil currently has more than 1000 surgeons affiliated with the society and skilled in general cardiovascular surgery. Many are adult surgeons who, during training, acquired some pediatric experience.

Following the recommendations of the European Association for Cardio-Thoracic Surgery Congenital Heart Disease Committee in the document “Optimal Structure of a Congenital Heart Surgery Department in Europe”, each surgeon should perform a minimum of 125 operations per year.

Considering the number of surgeons dedicated to congenital heart surgery in Brazil, we should have the capacity to perform at least 21,125 surgeries per year. On the other hand, the analysis of this data must be done with great caution.

A pediatric cardiac program needs a well-trained multidisciplinary team, not only surgeons. One of the biggest problems we face is the lack of pediatric ICU beds dedicated to PCS. Regardless of the type of hospital - a general or a pediatric hospital - a child needing cardiac surgery competes for the same bed with children who require intensive care for other illnesses. Hospitals with designated units for the postoperative care of patients with CHD perform more surgeries. Patients have shorter LOS and fewer mechanical ventilation days with lower costs when compared to those that share their beds with non-cardiac surgery children^[21]^. In our country, the lack of standardization of pediatric cardiac ICU care significantly impacts the number of procedures and their outcomes. In general, the scope of practice of the professional staff in these units follows the same rules as a pediatric ICU. Then, it is understandable that the complexity of pediatric patients who need ICU are generally less demanding and require a lower workload than a complex CHD child just operated on or in cardiogenic shock requiring mechanical circulatory support.

The number of dedicated cardiac ICU beds affects the volume of procedures performed in almost 80% of active centers in Brazil, but this is not the only limiting factor. In addition, the lack of specialized personnel such as nurses, pediatric cardiologists, and intensivists is a significant burden.

The lack of trained pediatric intensivists is not new, and it is also a widespread problem. Furthermore, there is an urgent need for high-quality cardiac intensive care programs to improve the quality of PCS services to reduce morbidity and mortality after surgery^[22]^.

Training of health workers in pediatric cardiac intensive care is vital before implementing a program in a limited resources setting. Cardiac critical care issues may affect a limited resource setting in many ways. In children, cardiopulmonary bypass (CPB) may impact multiple organ systems by modifying vasomotor tone, causing fluid shifts, and leading to pulmonary, kidney, gastrointestinal, neurologic, hematologic, and endocrine dysfunctions, increasing the risk of infections^[23]^.

In addition, a well-functioning multidisciplinary team requires specialized anesthesiologists, skillful nurses, and respiratory therapists who work together to handle pediatric cardiac patients and their families.

Suppose we combine highly specialized skills with a low payment that is inversely proportional to the workload. In that case, it is understandable that PCS is not attractive to most health professionals, especially those still in training. Conversely, it is common among pediatric cardiologists to look into further training in other subareas such as echocardiography and intervention, since these areas are better paid than cardiac hospitalists or intensivists. Usually, our clinicians dedicate time to intra-hospital consultations, follow-up on the wards, or concentrate at the hospital’s outpatient clinics. Therefore, our pediatric cardiac hospitals or specialized wards are filled with “part-time doctors” with parallel jobs and not working as part of a multidisciplinary team, as expected for a high-performance PCS program.

In summary, low salaries, demanding specialization, and excessive workload are known constraints to develop PCS of excellence. As pointed out by J. Tweddell: “Congenital heart surgery in limited resources environments will require visionary, innovative and dedicated individuals, and their commitment must be matched, fostered and encouraged with a leadership/administrative culture that recognizes the importance and potential of congenital heart surgery to both save lives and prevent suffering”^[24]^.

### F. International Cooperation: Moving Towards a New Scenario.

Because the world faces many daunting problems, we cannot expect to solve the maldistribution and poor access to cardiac surgery exclusively through the regular channels of international aid. However, numerous groups worldwide are involved with structured international projects without standardization and coordination^[25]^.

There are a limited number of programs involved in bringing patients to affluent countries for free cardiac surgery, which is not the case in Brazil. Most of them focus on developing a partnership between a recognized center of excellence from a developed country and a local host program. This partnership involves a visiting team, teaching, training, performing collaborative research, and donating equipment. The team usually includes surgeons, anesthesiologists, intensivists, cardiologists, perfusionists, interventional cardiologists, and nurses. This “twinning process” results in a transfer of knowledge, ideas, and skills. However, to avoid squandering energy and resources, it is essential to identify places, “fertile sites”, with receptive individuals where good work is already being done. This approach will be most effective when local governments, doctors, nurses, and hospital personnel have a genuine learning interest to solve their problems. Eventually, the host program will become autonomous, with the donor program assuming a consultant role^[26]^.

The international partnership has encouraged changes in medical practice, nursing empowerment, and leadership, helping to improve outcomes and quality in our centers. It is essential to mention two important partnerships presently acting in Brazil: the non-governmental organizations (NGOs) Children’s HeartLink (CHL) and The Boston Children’s Hospital initiative with The International Quality Improvement Collaborative (IQIC) for Congenital Heart Disease.

For over thirty-six years, CHL has been dedicated to prevent and treat children’s heart disease in developing countries. CHL works/partners with established/active cardiac programs devoted to pediatric charity care and the associated hospitals are operational year-round. There are some basic requirements for such partnership: the associated local institutions must track and measure outcomes and have local/provincial governmental support committed to CHL principles and collaboration. In addition, it should have the potential to become a regional cardiac center. CHL has supported the development of pediatric cardiac care in Brazil since 2009 at the Hospital da Criança e Maternidade in São José do Rio Preto, São Paulo. More recently, CHL established a partnership with two other Brazilian centers: Hospital de Messejana in Fortaleza (2015) and InCor, Faculdade de Medicina da Universidade de São Paulo (2018). Using the strategy of “twinning programs”, pairing two cardiac programs - one an established center of excellence, and the other an evolving program in a developing country -, CHL aims to establish a valuable relationship between both organizations. In Brazil, they count the volunteer training partners from Children’s Hospital in Minnesota (Minneapolis), Mayo Clinic (Rochester, Minnesota), and Seattle Children’s Hospital (Seattle) in the United States of America, and The Hospital for Sick Children (Toronto) in Canada.

Data published by Croti et al.^[27]^ shows that the partnership with CHL and monitoring with IQIC were fundamental for better communication, enhancement of teamwork, with a consequently significant reduction in morbidity and mortality. They also reported the partnership as a source of professional growth focused on increasing the quality of care for patients with CHD. The improvements portray the true essence of teamwork and their capacity for changing the culture at a Brazilian center.

The IQIC was officially launched in 2008 to provide benchmarking data for CHD surgery in the developing world to guide quality improvement efforts and reduce mortality. A significant contribution of the IQIC was local benchmarking, *i.e.*, using their solid database, participating centers from LMICs are now able to compare results with each other, on top of learning from quality assurance webinars given by the IQIC team. There are currently over 70 sites in more than 25 different countries - seven of them enrolled with the IQIC in Brazil. Furthermore, the program aims to create tailored quality improvement strategies to reduce mortality and major complications in emerging programs. For example, the partnership between CHL and São José do Rio Preto’s group, utilizing a seven-year analysis of the IQIC database, demonstrated structural and human flaws, whose resolution led to a significant decrease in infections and consequent reduction in mortality, despite an increase in the complexity and volume of their pediatric and CHD population.

By employing a telemedicine platform to facilitate distance learning, a series of webinars has helped enhance dialogue, disseminate knowledge and skills focusing on team training, nursing empowerment, and improvement of quality of care. One example of the importance of this collaboration was a Perfusion Course specially designed for all the participating centers. The aim of this project was ultimately to improve the quality of CPB for the IQIC member sites. The Boston Children’s perfusion team created ten perfusion practice webinars roughly based on the textbook previously published by the project coordinator^[28]^.

Such webinars are an efficient mean for distance learning for the 64 member institutions worldwide. Each webinar has been given on a defined schedule and recorded to allow partner centers to view them live or at their convenience. Live presentations included questions and answers sessions. This perfusion education series remains available online for IQIC member institutions.

Moreover, with the support of the NGO Milagros para Niños, the Boston Children’s Hospital has invited 15 perfusionists from Latin America to visit their program for one week to observe perfusion practices and the cardiac operating room team dynamic. These visits were structured to maximize exposure to perfusion practices during their stay, with a dedicated Spanish/Portuguese translator facilitating the communication with Boston’s perfusion team. These collaborative efforts have culminated with a publication in the Brazilian Journal of Cardiovascular Surgery which recommends a phased adoption of the American Society of Extracorporeal Technology’s Standards and Guidelines^[7]^ in Brazil. The Standards and Guidelines for Perfusion Practices of the SBCCV and the Sociedade Brasileira de Circulação Extracorpórea (or SBCEC) have been published as a resource to those pediatric cardiac programs seeking to improve their outcomes, being a reference for good practices^[12]^.

The IQIC and the Boston Children’s Hospital perfusion teams are uniquely positioned to collaborate with international programs to share ideas to improve outcomes in patients with CHD requiring CPB.

Another example of a partnership for improving Brazilian PCS was the collaboration between the InCor, of the Universidade de São Paulo, and the Penn State Health Center for Pediatric Cardiovascular Research. The translational research among cardiac surgery centers, countries, and continents stands to improve the clinical outcomes for thousands of patients. This research, a “bench-to-bedside and beyond” approach, aims to improve individual and public health by generating multicenter and multidisciplinary collaboration to pull discoveries from basic science arising from laboratory, clinical, or population studies into clinical applications^[29]^.

Brazil manufactures many medical devices approved by the Agência Nacional de Vigilância Sanitária (or Anvisa) available only in this region, without further clinical data or benchmarking with other significant research centers developing similar devices^[30]^. Usually, only Food and Drug Administration-approved products in widespread use in developed countries are research objects by the international scientific community. This collaboration has improved clinical outcomes for pediatric cardiovascular patients in Brazil and other countries using the concept of translational research, modifying our practice by evidence-based research publications rather than dogmas. The choice of circuits for performing PCS with CPB is an example of this approach^[29^,^31^-^33]^.

Other numerous initiatives are assisting Brazil to improve the quality of care for better outcomes, such as the World Congenital e Pediatric Surgery Database, which has been helping more centers by interpreting their local data and finding ways to be better. The series of webinars launched either by the World Congenital and Pediatric Cardiac Surgery Society or the Congenital Heart Academy promotes democratization in scientific knowledge via complimentary online access. This new way of knowledge dissemination worldwide represents an affordable way for health care professionals to improve and learn in a country like ours.

### G - Advancing in the Face of a Controversial Dilemma: How to Focus on Quality When Access Is Still an Issue?

Considering the importance of CHD in the composition of Infant Mortality Rate (IMR) in wealthier countries, improving care in this area has significantly impacted overall mortality. Hence, PCS could have a very positive impact on the IMR. Therefore, a comprehensive program caring for children with CHD through cardiac surgery is a worthy public policy and one that should concern our government.

Many LMICs struggle to provide the investment required by the health services for lifesaving CHD. A recent publication show correlations between various development indicators and CHD surgery mortality^[34]^. Nonetheless, there is variation among countries of similar GDP per capita levels. Finding lower risk-adjusted mortality in some countries with lower levels of development offers hope and encourages investment in CHD surgery programs.

Translating it to Brazil, access to surgical services and reduction of CHD mortality are crucial to impact IMR. Once again, the abovementioned publication demonstrated a link between various development indicators and CHD surgery mortality, calling for more investigation at a regional level to improve the quality of care and increase access by encouraging investment in congenital heart surgery programs and subspecialty training.

Outcomes matter, especially in places where the economic burden is a constraint. Transparency with the outcomes data from all pediatric cardiac centers in Brazil is mandatory to evaluate and create a solid network caring for patients with CHD. We know that talking directly about results and problems is risky because most people do not appreciate such discussions. However, it is essential to evaluate outcomes, understand the local constraints, and plan solutions. Although enumerating the problems may be easy, finding the answer is increasingly tricky in settings without political stability and sustainable economic growth. Centers of excellence, with proper funding and workforce for research to generate new knowledge, particularly in children and adults with CHD, are needed for more efficient treatment, improved cost-benefit ratio, and sustainability of care. These centers can then disseminate the new knowledge, minimizing or eliminating the learning curve and developing policies for the specialty’s future.

Collaborative quality improvement programs have improved health care quality in many different scenarios because they help target reasons for such variations and find solutions for shared problems. Joining a coordinated quality improvement program has proved beneficial in other countries^[35^-^37]^.

With this in mind, an important initiative was developed in 2017, creating a consortium between centers of PCS in the State of São Paulo called ASSIST^[38]^. It was the first Brazilian initiative to build a collaborative quality improvement program in pediatric cardiology. The ASSIST was initially funded by the Brazilian Ministry of Health, the Conselho Nacional de Desenvolvimento Científico e Tecnológico (or CNPq) and the São Paulo State, all through the Fundação de Amparo à Pesquisa do Estado de São Paulo (or FAPESP). ASSIST was a notable milestone in developing a collaborative data-sharing network between participating centers, initiating multicenter studies in CHD. Although it has not yet reached all Brazilian centers, this database was critical in evaluating costs in PCS in our country, helping to update the reimbursement tables for hospitals in the health system. More recently, another significant contribution published in Brazilian scientific journals, with international experts’ collaboration, deserves to be highlighted: the Translation and Validation of the Boston Technical Performance Score (TPS) in a Developing Country^[39]^. The authors showed their initial experience using TPS translated to Portuguese for their congenital cardiac surgical team’s performance analysis. The findings in 972 surgeries strengthen pediatric and congenital cardiac surgeons’ technical accuracy role as a predictor of adverse outcomes after adjusting for other covariates. Patients assigned as “inadequate surgical result” had 3.2 times more chance of death and 1.8 times more complications. Objective evaluation of residual defects associated with data collected with the ASSIST database could adjust current surgical results, decreasing mortality and morbidity through better clinical practices.

Those institutions in which PCS is combined with adult programs need to understand the differences in the business plan, clinical pathways, workforce, mindset, training, different technology needs, and infrastructure of both disciplines. Before signing up, each group’s managers should clearly understand what they want from the alliance. Most important is gaining support at all levels of each domain for working together.

In summary, the importance of sharing data and establishing regional and national registries cannot be understated. A culture of data-driven decision-making should be the norm, and local institutions, academic centers, medical societies, and ultimately the payer system (the government) should support such initiatives. Only outcomes evaluation in a systematic fashion can delineate standards of care, improve survival, decrease morbidity, and enhance the quality of life for children with CHD. Also, guidelines and structured outcomes evaluation will create gold standards of care, consequently improving outcomes.

## CONCLUSION

PCS progress is still the result of personal efforts, dedication, and personal resilience. International partnership programs focusing on the quality of outcomes have the potential to bring a new dawn to Brazil if associated with adequate local support and leadership. Low payment, work overload, and complexity of care appear as a constraint to increasing interest, augmenting the number of specialized centers and consequently the number of PCS procedures. The role of governments, NGOs, or other stakeholders in the training and retention of skilled professionals should be seriously addressed to build a robust health system to care for CHD patients.

We do not yet have the clout to advocate for our “orphan” pediatric cardiovascular services, particularly without a collective approach. Therefore, it is crucial to determine what should be preserved, what needs improvement, how it can be done, by whom, and what we must transform.

There is much ado, and it is a task not exclusive to surgeons. Since PCS is a complex system with multiple components that demand teamwork and the authorities’ active participation that control the power, a creative and collective approach is required.

**Table:** 

Authors’ Roles & Responsibilities	
LFC	Substantial contributions to the conception or design of the work; or the acquisition, analysis, or interpretation of data for the work; drafting the work or revising it critically for important intellectual content; final approval of the version to be published
LAM	Substantial contributions to the conception or design of the work; or the acquisition, analysis, or interpretation of data for the work; drafting the work or revising it critically for important intellectual content; final approval of the version to be published
DG	Substantial contributions to the conception or design of the work; or the acquisition, analysis, or interpretation of data for the work; drafting the work or revising it critically for important intellectual content; final approval of the version to be published
RN	Substantial contributions to the conception or design of the work; or the acquisition, analysis, or interpretation of data for the work; drafting the work or revising it critically for important intellectual content; final approval of the version to be published
